# Pharmaceutical Strategies for Translating Klotho-Based Therapeutics: From Biologic Developability to Advanced Delivery Systems

**DOI:** 10.3390/pharmaceutics18091124

**Published:** 2026-09-07

**Authors:** Lingxin Zeng, Xuan Chen, Ying Li, Wei Xiong

**Affiliations:** School of Pharmacy, Shenzhen University Medical School, Shenzhen University, Shenzhen 518055, China; a13411148686@163.com (L.Z.); xuanchen0728@163.com (X.C.); li.ying@szu.edu.cn (Y.L.)

**Keywords:** Klotho, developability, drug delivery, translational pharmacology, formulation

## Abstract

Klotho is a longevity-associated and tissue-protective protein involved in mineral metabolism, oxidative stress, inflammation, fibrosis, cellular senescence, and neurovascular homeostasis. However, nearly three decades after its discovery, no Klotho-based therapy has been approved, highlighting a translational gap that extends beyond biological validation. This review reframes Klotho translation as a pharmaceutical sciences challenge, focusing on how to convert Klotho into a druggable, manufacturable, deliverable, and clinically controllable therapeutic product. We summarize the isoform-specific properties of membrane-bound α-Klotho, soluble α-Klotho, and β-Klotho that are relevant to product design, and review the current clinical and preclinical landscape dominated by gene-, mRNA-, and antibody-based approaches. We further distinguish confirmed developability barriers, including renal handling and limited systemic persistence, from plausible risks common to macromolecular biologics, such as aggregation, chemical degradation, immunogenicity, and poor tissue penetration. Finally, we evaluate emerging delivery and formulation strategies, including viral and non-viral gene delivery, extracellular vesicles, hydrogels, ultrasound-targeted microbubbles, osmotic pumps, and long-acting protein engineering. An integrated roadmap combining molecular engineering, disease-specific delivery, pharmacokinetic/pharmacodynamic biomarkers, manufacturability assessment, and repeated-dose safety evaluation may help transform Klotho from a promising anti-aging molecule into a clinically viable biologic platform.

## 1. Introduction

Klotho was first identified in 1997 as a gene whose disruption caused a syndrome resembling accelerated aging in mice, whereas Klotho overexpression extended lifespan in experimental models [1,2]. Since then, Klotho biology has expanded from an aging phenotype to a broad network of endocrine, renal, cardiovascular, metabolic, and neuroprotective functions [3,4]. The Klotho family primarily consists of two distinct isoforms, α-Klotho and β-Klotho, which differ in tissue distribution, ligand preference, and physiological functions [3]. α-Klotho participates in FGF23-dependent mineral metabolism and exerts soluble, pleiotropic actions in multiple tissues, whereas β-Klotho functions primarily as a co-receptor for endocrine FGF19/FGF21 signaling and is central to metabolic regulation [5,6,7]. This mechanistic breadth has made Klotho one of the most attractive biomolecules in aging-related therapeutics.

The paradox is that strong biological rationale has not yet translated into an approved Klotho-based medicine. The literature contains abundant evidence of benefit in models of kidney injury, vascular calcification, cardiac remodeling, neurodegeneration, fibrosis, and metabolic dysfunction [8,9,10,11,12,13]. Nevertheless, the visible clinical pipeline remains sparse and early. Public records and company disclosures now include non-viral Klotho plasmid programs, a β-Klotho-targeting antibody agonist, a Klotho AAV-oriented program for amyotrophic lateral sclerosis, an α-Klotho protein formulation intended for cognitive impairment, and an α-Klotho mRNA/LNP program in healthy adult volunteers (Table 1). These efforts indicate that the field is entering clinical translation, but they also reveal the absence of a mature consensus on the optimal modality, route, target tissue, dosing interval, and safety monitoring strategy.

This review assumes, in line with recent expert opinion, that the bottleneck in Klotho translation is increasingly pharmaceutical rather than purely biological [14]. In other words, the key question is not only whether Klotho is protective, but how Klotho can be manufactured reproducibly, remain structurally and functionally stable, reach the correct tissue at the correct exposure level, avoid unacceptable immune or metabolic risk, and be administered in a way compatible with chronic disease management. This perspective differs from conventional Klotho reviews that emphasize signaling pathways and disease mechanisms. Here, we focus on developability, delivery, formulation, and translational product design.

**Table 1:** Overview of publicly disclosed Klotho-related therapeutic programs and their translational implications.

Program/Agent	Modality	Indication	Public Stage	Key Translational Point
NCT07216781/Minicircle	Non-viral Klotho plasmid/minicircle gene therapy	Healthy adult volunteers; cognition and health-related measures	Phase 1 record listed publicly [15,16]	Illustrates the move toward body-as-bioreactor expression rather than repeated recombinant protein dosing.
JMT202/CSPC Pharmaceutical Group Limited	Recombinant fully human anti-β-Klotho monoclonal antibody; FGFR1c/β-Klotho receptor agonist	Hypertriglyceridemia; broader metabolic disease potential	FDA clinical-trial approval in the U.S.; NMPA approval obtained in May 2024 with trials in China [17]	β-Klotho is used as a receptor-associated metabolic target rather than as protein replacement.
KLTO-202/Klotho Neurosciences	Secreted-Klotho promoter/gene/delivery system; AAV-oriented program	Amyotrophic lateral sclerosis	FDA Orphan Drug Designation reported in July 2025 [18,19]	Highlights tissue-selective expression and neuromuscular delivery as alternatives to systemic protein injection.
JN-0413/Jocasta Neuroscience	Proprietary α-Klotho protein formulation	Cognitive impairment associated with neurodegenerative disease	Preclinical; IND submission publicly stated as planned for Q4 2026 [20]	One of the few publicly visible efforts centered on an α-Klotho protein formulation.
NCT07544420/Klothea Bio	α-Klotho mRNA formulated in lipid nanoparticles	Healthy adult volunteers/longevity-associated biomarkers	Phase 1b clinical study record and public launch announcement [21]	Represents a potentially repeatable expression platform, but requires careful dose control and biomarker validation.

## 2. Biological Functions of Klotho

### 2.1. Klotho Isoforms

**Figure 1:**
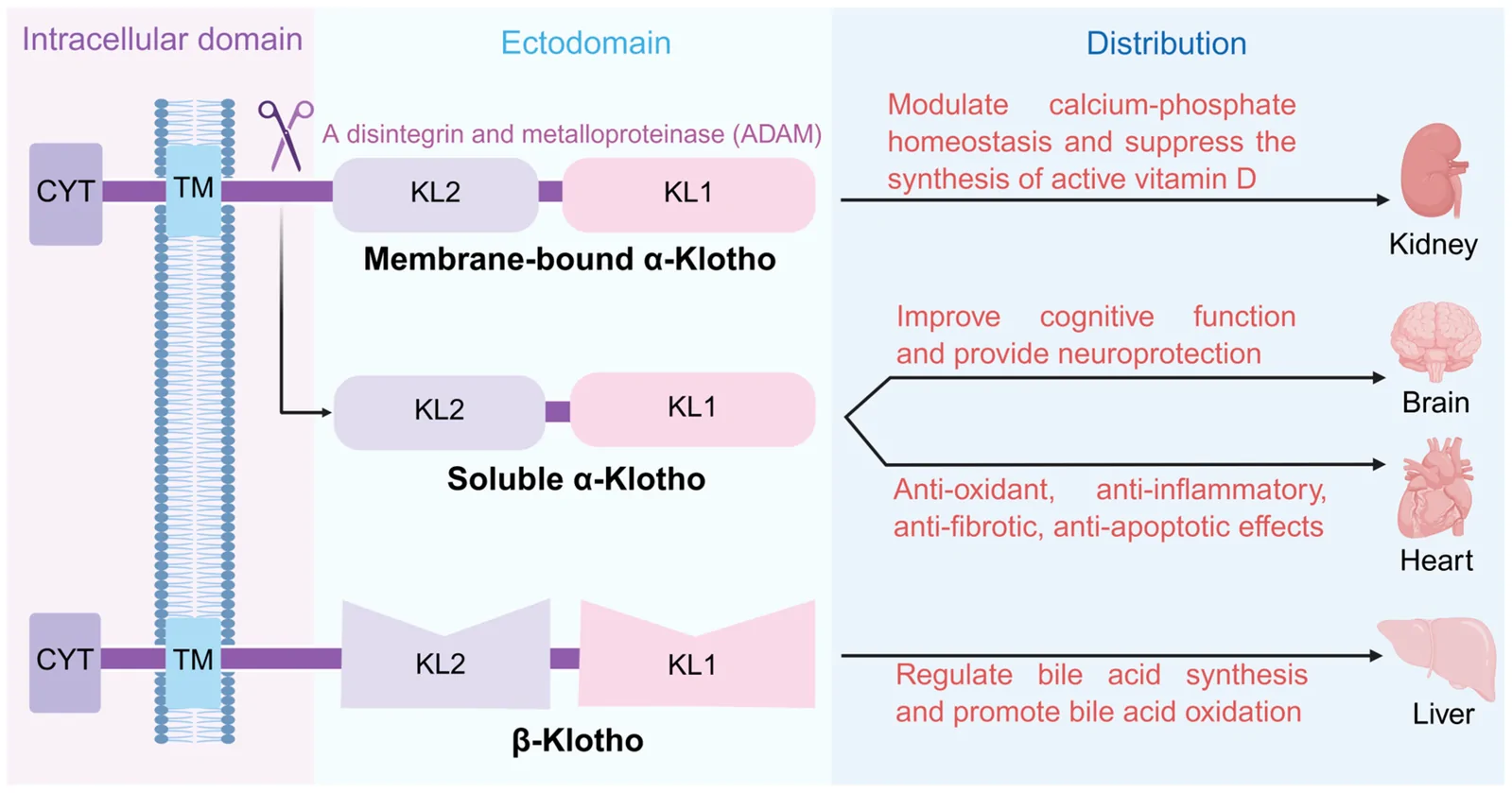
Schematic representation of Klotho isoforms, their structural domains, and tissue distribution. The main Klotho isoforms include membrane-bound α-Klotho, soluble α-Klotho, and β-Klotho. Domain structures (KL1, KL2) and the a disintegrin and metalloproteinase (ADAM) cleavage site are indicated. Subcellular localization, tissue distribution, and representative biological functions are summarized for each isoform. Created with BioRender.com.

The Klotho family is commonly discussed in terms of α-Klotho and β-Klotho (Figure 1). α-Klotho is expressed primarily in the kidney and choroid plexus and exists as a membrane-bound form and soluble forms generated by ectodomain shedding or alternative processing [3,4,22,23]. Membrane-bound α-Klotho acts as an essential co-receptor for FGF23, enabling FGF23-FGFR signaling that regulates phosphate excretion and vitamin D metabolism [13,24,25]. Soluble α-Klotho is a circulating or paracrine factor with broader biological activities, including modulation of oxidative stress, inflammatory signaling, ion channel stability, fibrosis, and cellular stress responses [11,12,26,27,28]. Notably, soluble forms are generated via two distinct mechanisms: ectodomain shedding by ADAM proteases (primarily ADAM10 and ADAM17) at the cell surface, and alternative splicing of the KL transcript, which yields a secreted isoform lacking the transmembrane and cytoplasmic domains. Although both forms circulate and share core KL1-KL2 homology, they may not be functionally equivalent. Recent quantitative analyses indicate that shed Klotho is the predominant circulating species in both mice and humans [23], whereas the spliced variant is expressed at substantially lower levels under physiological conditions [29].

These isoforms imply different therapeutic modalities. Membrane-bound α-Klotho is difficult to replace directly as a recombinant protein because membrane localization and receptor complex formation are essential to its canonical endocrine function. It is therefore more naturally suited to gene, cell, or membrane-associated vesicle strategies. Soluble α-Klotho, by contrast, is conceptually compatible with recombinant protein therapy, mRNA expression, gene delivery, or sustained-release formulation. β-Klotho differs from both because it is primarily a co-receptor for endocrine FGF19/FGF21 signaling, making it attractive as a receptor-associated target for agonistic antibodies or FGF analogs rather than as a simple replacement protein [5,6,7].

### 2.2. Pharmacological Activities and Safety Concerns

α-Klotho exerts protective effects through several overlapping mechanisms (Table 2). In the kidney, the FGF23-α-Klotho axis regulates phosphate and vitamin D homeostasis, which is directly relevant to vascular calcification and chronic kidney disease [10,13,25,30]. Soluble α-Klotho also enhances antioxidant defenses, partly through Nrf2 and FoxO-related pathways, and suppresses inflammatory programs such as TLR4/NF-κB signaling [26,27,31,32,33]. In fibrotic disease models, soluble α-Klotho antagonizes TGF-β/Smad and Wnt/β-catenin signaling, thereby limiting myofibroblast activation and extracellular matrix deposition [11,34,35,36]. α-Klotho has also been linked to anti-apoptotic, anti-pyroptotic, autophagy-modulating, cardiovascular, and neuroprotective effects [10,37,38,39,40,41,42,43,44,45].

These activities are therapeutically attractive but also create safety and exposure challenges. α-Klotho is not a single-pathway inhibitor; it is a pleiotropic endocrine and paracrine regulator. Systemic overexpression or prolonged exposure may therefore influence phosphate balance, calcium handling, vitamin D metabolism, insulin/IGF-related signaling, and vascular biology [3,4,5,36]. A therapeutic product should thus be designed not merely to maximize soluble α-Klotho exposure, but to provide controlled exposure within a pharmacologically effective and physiologically tolerable window.

On the other hand, β-Klotho biology raises a different set of considerations (Table 2). As a co-receptor for FGF19 and FGF21, β-Klotho integrates bile acid metabolism, glucose and lipid handling, energy expenditure, and hepatic homeostasis [5,6,7,46]. Antibody or ligand-based activation of β-Klotho-associated receptor complexes may offer metabolic benefits, but the development logic is closer to receptor pharmacology than to α-Klotho replacement. For this reason, α-Klotho replacement and β-Klotho receptor-targeting products should be compared in a clinical pipeline table but not conflated mechanistically.

**Table 2:** Biological and pharmacological properties of major Klotho isoforms.

Klotho Isoform	Main Structural Features	Major Ligands	Tissue Distribution	Biological Functions	Therapeutic Implication
Membrane-bound α-Klotho	Type I transmembrane protein containing KL1 and KL2 extracellular domains; can be cleaved by ADAM proteases to generate soluble forms [47]	Acts as an essential co-receptor for FGF23 signaling through FGFR1c, FGFR3c, and FGFR4 [3]	Highly expressed in kidney and choroid plexus	Regulates phosphate excretion, calcium balance, and vitamin D metabolism; contributes to mineral homeostasis and suppression of ectopic calcification	Direct recombinant replacement is difficult because membrane localization is required; more suitable for gene-, cell-, or membrane-associated delivery strategies [3]
Soluble α-Klotho	Circulating or paracrine form generated by ectodomain shedding or alternative processing; contains KL1/KL2-related functional domains	May modulate FGF23-related signaling and multiple FGF23-independent pathways	Circulation, kidney-associated compartments, brain-related compartments, cardiovascular and other peripheral tissues	Anti-oxidative, anti-inflammatory, anti-fibrotic, anti-apoptotic, cardiovascular protective, renal protective, and neuroprotective effects	Most compatible with recombinant protein, engineered peptide, fusion protein, mRNA, gene delivery, EV-based delivery, or sustained-release formulation [8,48]
β-Klotho	Single-pass transmembrane co-receptor structurally related to α-Klotho but functionally distinct	Co-receptor for endocrine FGF19 and FGF21 signaling, especially through FGFR1c and FGFR4 complexes [49,50]	Metabolic tissues, especially liver, adipose tissue, and related endocrine-metabolic organs	Regulates bile acid metabolism, glucose and lipid handling, energy expenditure, thermogenesis, and hepatic metabolic homeostasis	Better viewed as a receptor-associated metabolic target; β-Klotho-targeting antibodies or FGF analogs should not be conflated with α-Klotho replacement therapy [5,6]

## 3. Current Translational Landscape of Klotho-Related Therapeutics

The publicly visible translational landscape of Klotho-related therapeutics remains early, fragmented, and modality-driven rather than indication-driven (Table 1). Current programs do not yet converge on a single optimal therapeutic format. Instead, they span non-viral plasmid gene therapy, mRNA/LNP-mediated α-Klotho expression, AAV-oriented soluble α-Klotho expression systems, proprietary α-Klotho protein formulations, and β-Klotho-targeting agonistic antibodies. Public clinical records list a non-viral Klotho plasmid program in healthy adult volunteers and an α-Klotho mRNA/LNP Phase 1b study designed to assess safety and tolerability in healthy adults. In parallel, company disclosures and industry reports describe a β-Klotho-targeting monoclonal antibody program for hypertriglyceridemia, an AAV-oriented secreted-Klotho program for amyotrophic lateral sclerosis, and a proprietary α-Klotho protein formulation being advanced for cognitive impairment associated with neurodegenerative disease.

This diversity indicates that Klotho translation is not simply a matter of selecting an indication, but of matching a biological hypothesis with a feasible product architecture. Broadly, the current landscape can be divided into three translational categories. The first category is α-Klotho restoration by endogenous expression, including plasmid, viral-vector, and mRNA-based approaches. These strategies attempt to bypass some limitations of recombinant protein administration by turning host tissues into transient or sustained sources of Klotho. Their potential advantages include prolonged exposure, reduced injection frequency, and the possibility of tissue-directed expression. However, they shift the central challenge from protein formulation to expression control, tissue distribution, immune response, reversibility, and long-term safety monitoring. For aging-related or preventive indications, this shift is particularly important because long-duration expression may be difficult to justify unless the exposure window, stopping rules, and safety biomarkers are well defined.

The second category is direct α-Klotho protein replacement or protein-based formulation. This is conceptually the most straightforward approach because it resembles conventional biologic therapy: the active molecule is manufactured, characterized, dosed, and monitored as a defined pharmaceutical product. Compared with gene- or mRNA-based approaches, recombinant or soluble α-Klotho protein formulations may offer better dose titration, reversibility, and chemistry, manufacturing, and controls (CMC) controllability. However, this modality appears underrepresented in the visible pipeline, likely reflecting unresolved barriers related to protein half-life, conformational stability, glycosylation heterogeneity, aggregation risk, repeated dosing feasibility, and large-scale manufacturability. Therefore, the scarcity of clinical protein-replacement programs should not be interpreted as weak biological rationale, but rather as evidence that native or near-native α-Klotho remains a difficult biologic product to develop.

The third category is β-Klotho receptor pharmacology, represented by agonistic antibodies targeting β-Klotho-containing receptor complexes. This modality should be distinguished mechanistically from α-Klotho replacement. β-Klotho functions primarily as a co-receptor in endocrine FGF signaling, especially within the FGF19/FGF21 metabolic axis. Thus, β-Klotho-targeting antibodies are closer to receptor agonists or pathway modulators than to Klotho replacement therapies. Their translational endpoints are also different: lipid lowering, insulin sensitivity, hepatic metabolism, bile acid biology, and cardiovascular safety are more relevant than restoration of soluble α-Klotho deficiency. For this reason, α-Klotho- and β-Klotho-related products may be compared within the same landscape table, but they should not be treated as a single mechanistic class.

Several implications emerge from this landscape. Firstly, expression-based strategies appear more prominent than direct soluble α-Klotho protein replacement, suggesting that current developers are attempting to overcome pharmacokinetic and formulation limitations by changing the mode of Klotho production. However, these platforms shift rather than remove translational risk, creating new concerns related to vector design, tissue tropism, expression duration, dose control, and inter-individual variability. Secondly, Klotho-related programs are expanding beyond conventional disease treatment toward healthy aging, cognition, and health span-associated biomarkers. This trend raises the evidentiary bar, particularly for safety, reversibility, biomarker validity, and clinically meaningful endpoints.

Accordingly, modality selection should be indication-specific. Longer-duration gene expression may be more acceptable in severe progressive diseases, whereas transient and controllable platforms are preferable for preventive or longevity-oriented applications. β-Klotho-targeting programs should also be evaluated separately as chronic metabolic receptor-modulating therapies rather than as α-Klotho replacement. Overall, the current pipeline demonstrates that Klotho biology is entering translation, but it also highlights unresolved choices regarding protein versus expression platforms, systemic versus local delivery, transient versus sustained exposure, and α-Klotho restoration versus β-Klotho receptor modulation.

## 4. Developability Barriers: Separating Evidence from Inference

A major weakness in the current field is the tendency to discuss Klotho primarily as a biologically active molecule without defining its product-critical quality attributes. For a biologic, therapeutic activity is only one component of translatability. A clinically viable Klotho product must also have predictable pharmacokinetics, acceptable stability, reproducible potency, manageable immunogenicity risk, scalable manufacturability, feasible administration route, and batch-to-batch comparability. This distinction is particularly important for Klotho because the term “Klotho therapy” may refer to several different product types, including recombinant soluble α-Klotho, engineered Klotho fragments, fusion proteins, mRNA/LNP systems, plasmid or viral-vector expression platforms, extracellular vesicles, and β-Klotho-targeting antibodies. Each modality carries a different developability profile and should not be evaluated using the same assumptions.

Among Klotho-specific barriers, the strongest direct evidence concerns renal handling and systemic persistence (Table 3). Hu et al. demonstrated that the kidney plays dual roles in α-Klotho homeostasis, contributing to both its production and clearance, and that exogenously administered α-Klotho persists longer in anephric rats than in normal controls [51]. Complementing these findings, Zhong et al. provided quantitative pharmacokinetic evidence that the physicochemical properties of recombinant α-Klotho can also markedly influence systemic exposure. Following intravenous administration of CHO-derived soluble α-Klotho to rats at 0.5 mg/kg, the reported terminal half-life, systemic clearance, and steady-state volume of distribution were 12.2 h, 15.8 mL/h/kg, and 120 mL/kg, respectively [52]. By contrast, HEK293-derived soluble α-Klotho was cleared so rapidly that systemic exposure became undetectable beyond 5 min after dosing. This striking difference was attributed to distinct glycosylation profiles: HEK-derived soluble α-Klotho carries rare LacdiNAc (GalNAcβ1–4GlcNAc) N-glycan structures that may promote rapid hepatic clearance through asialoglycoprotein or mannose receptors, whereas CHO-derived soluble α-Klotho bears conventional sialylated complex-type N-glycans associated with slower clearance kinetics [52]. These findings underscore that both renal handling and production-dependent glycosylation can substantially shape the in vivo pharmacokinetic behavior of recombinant Klotho, rather than representing merely manufacturing-related variables.

Taken together, these observations have several important translational implications. First, renal function may be a major determinant of α-Klotho exposure, particularly in patients with chronic kidney disease or acute kidney injury and in aging populations, in whom endogenous Klotho homeostasis is already altered. Second, dose-exposure relationships may differ substantially between healthy individuals and patients with impaired renal clearance. Third, systemic α-Klotho therapy may therefore require disease-specific pharmacokinetic/pharmacodynamic modeling rather than simple body-weight-based dosing. Nevertheless, comprehensive human pharmacokinetic/pharmacodynamic data remain limited, particularly with respect to absorption, distribution, clearance, tissue exposure, exposure-response relationships, and accumulation following repeated administration.

A second evidence gap concerns pharmacodynamic measurement (Table 3). Klotho has pleiotropic effects on mineral metabolism, oxidative stress, inflammation, fibrosis, vascular biology, and neuroprotection. This breadth is biologically attractive but creates a major challenge for potency assay design. A useful Klotho potency assay should not merely detect the presence of the protein, but demonstrate preserved biological function in a pathway relevant to the intended indication. For kidney or vascular calcification indications, FGF23-related signaling, phosphate handling, and vitamin D metabolism may be relevant readouts. For fibrosis, inhibition of TGF-β/Smad or Wnt/β-catenin signaling may be more informative. For neurodegenerative or cognitive indications, oxidative stress, synaptic function, or neuroinflammatory markers may be needed. Without indication-specific potency assays, it will be difficult to compare different Klotho constructs, delivery platforms, or manufacturing batches.

Other limitations are plausible but not yet fully proven for Klotho (Table 3). Like many recombinant proteins, soluble α-Klotho may be vulnerable to aggregation, interfacial adsorption, oxidation, deamidation, fragmentation, glycation, and conformational changes during manufacturing, storage, transport, or administration [53,54,55,56,57]. These risks warrant systematic investigation for Klotho, particularly given its size, glycosylation, and multidomain architecture, all of which are known to influence the developability of protein therapeutics. Structural heterogeneity, aggregates, host-cell impurities, or non-native glycosylation could alter receptor engagement, tissue distribution, clearance, and anti-drug antibody formation [58,59]. Accordingly, recombinant or engineered α-Klotho products would benefit from early and systematic evaluation of stress stability, glycosylation profiles, subvisible particles, stability-indicating potency, and immunogenicity risk, even if the full extent of these risks remains to be established for Klotho specifically. Manufacturability should also be treated as an early translational barrier: full-length α-Klotho requires reproducible production of properly folded and glycosylated protein, whereas fragments, peptides, fusion proteins, gene therapies, and mRNA/LNP platforms introduce distinct CMC challenges related to functional completeness, receptor selectivity, vector or LNP quality, transgene integrity, expression consistency, and residual impurities.

**Table 3:** Confirmed and inferred developability barriers for Klotho therapeutics.

Developability Barrier	Current Evidence Status	Klotho Product Design Implications	Recommended Experiments
Short or variable systemic persistence	Partly confirmed; renal production, uptake, and clearance have been demonstrated for circulating α-Klotho [29,51].	Repeated dosing, sustained release, or expression-based approaches may be required.	Cross-species pharmacokinetics/pharmacodynamics, disease-state pharmacokinetics, renal impairment models, bioactive exposure assays.
Limited tissue penetration and BBB delivery	Inferred from macromolecule size and hydrophilicity; direct Klotho delivery data remain limited [29].	Systemic injection may be insufficient for CNS indications without targeted routes or expression strategies.	Tissue biodistribution, cerebrospinal fluid/plasma ratio, receptor-mediated transport, intranasal or vector-based delivery studies.
Aggregation and interfacial instability	Inferred from protein therapeutic class behavior [54,55,56,57].	May reduce potency and increase immunogenicity; critical for prefilled syringes and long-term storage.	SEC-MALS, DLS, micro-flow imaging, agitation/freeze–thaw/light-stress studies, receptor-binding assays after stress.
Chemical degradation	Inferred; Klotho-specific oxidation, deamidation, and fragmentation data are insufficient.	May affect receptor binding and potency; requires formulation screening.	LC-MS peptide mapping, forced degradation, glycan profiling, potency-stability correlation.
Immunogenicity	Inferred from recombinant protein and aggregate biology [58,59].	Repeated dosing in chronic disease could generate neutralizing or non-neutralizing ADAs.	In silico T-cell epitope screening, in vitro immune assays, ADA/NAb assays in repeat-dose animal studies.
Physiological on-target safety	Biologically plausible and partly supported by Klotho roles in mineral and metabolic regulation [3,4,5].	Dose escalation should monitor phosphate, calcium, vitamin D, FGF23, glucose/insulin, vascular markers.	Integrated safety pharmacology, mineral metabolism panels, exposure-safety modeling.

## 5. Current Klotho Delivery and Formulation Strategies

Delivery and formulation strategies for Klotho should be evaluated according to the translational problems they are designed to solve, rather than by carrier type alone. Because Klotho can be developed as a recombinant soluble protein, an encoded transgene, an mRNA-expressed product, a vesicle-associated cargo, or a locally released biologic, the delivery system is not simply an auxiliary carrier but a central component of product design.

**Figure 2:**
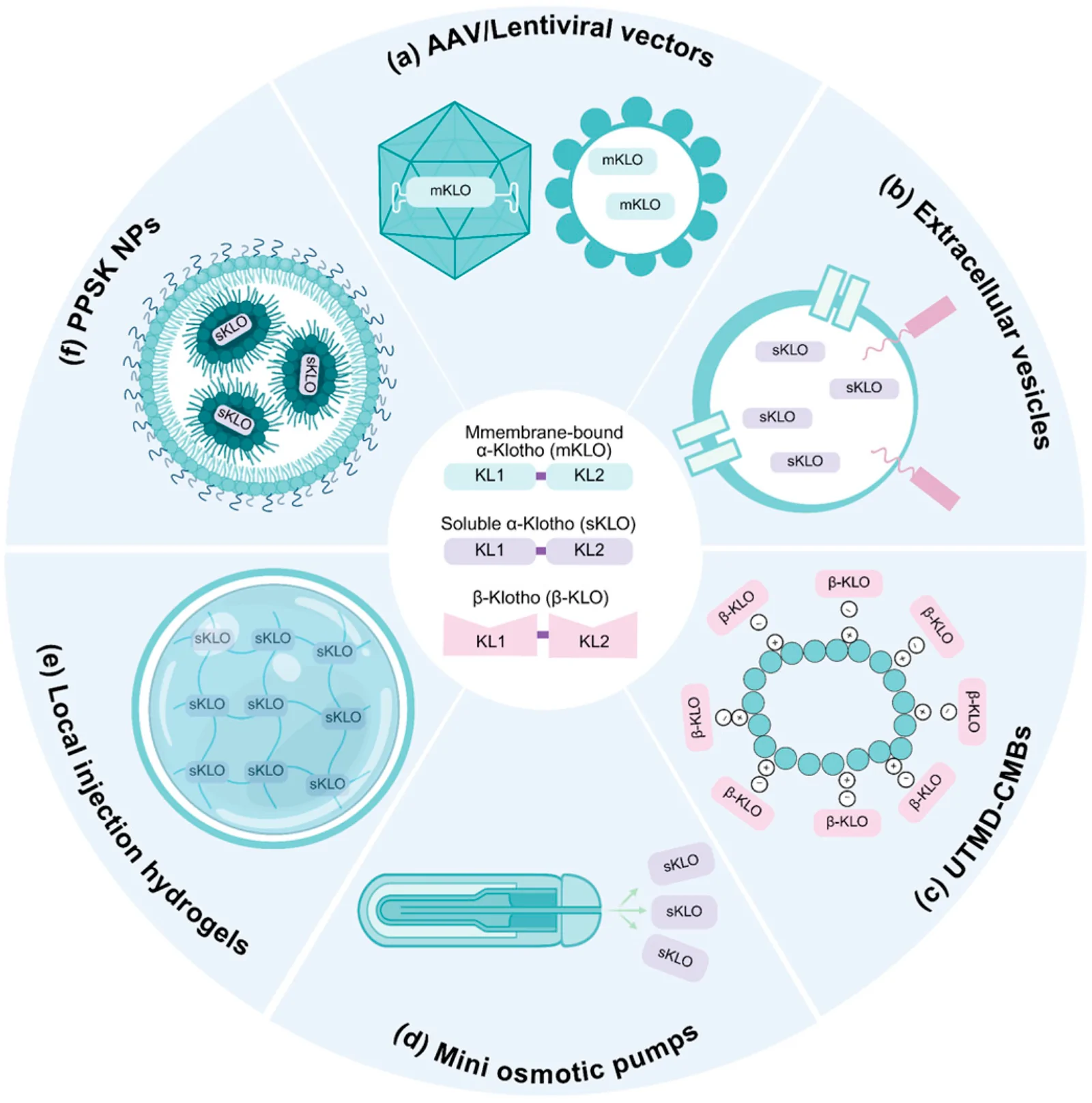
Representative delivery and formulation strategies for Klotho-based therapeutics. (**a**) AAV/lentiviral vectors enable sustained transgene expression and are useful for maintaining longer-term Klotho exposure in experimental disease models. (**b**) Extracellular vesicles provide biologically compatible vesicular transport for soluble or vesicle-associated Klotho and may facilitate renal tissue repair. (**c**) UTMD-CMBs combine ultrasound-targeted microbubble destruction with cationic microbubbles to support spatially guided cardiac gene delivery. (**d**) Mini osmotic pumps allow continuous systemic infusion of recombinant soluble α-Klotho, providing stable exposure for pharmacological proof-of-concept studies. (**e**) Local injection hydrogels retain Klotho at regional disease sites and enable sustained or programmable local protein release. (**f**) PPSK NPs represent kidney-targeted non-viral plasmid nanoparticles designed to deliver Klotho genes to injured renal tubular epithelial cells. Created with BioRender.com.

Current strategies can be broadly grouped into expression-based platforms, extracellular vesicle and cell-derived systems, recombinant protein delivery with sustained-release formulations, and route-oriented approaches for improving clinical usability. Representative examples are summarized in Table 4 and Figure 2.

### 5.1. Expression-Based Platforms: Viral Vectors, Non-Viral Plasmids, and mRNA/LNP Systems

Expression-based strategies aim to use host tissues as endogenous sources of Klotho, thereby reducing dependence on frequent recombinant protein administration. Viral vectors, including AAV and lentiviral systems, have demonstrated proof-of-concept activity in preclinical kidney and brain models [60,61]. Their main advantage is sustained expression, which is useful for testing whether long-term Klotho restoration can modify chronic pathological processes such as diabetic kidney injury, renal fibrosis, oxidative stress, and aging-related neurological dysfunction. These platforms are therefore most relevant when prolonged exposure is biologically justified, particularly in severe or progressive diseases. However, their translational value depends on tissue tropism, dose control, immunogenicity, and the ability to avoid excessive or irreversible Klotho expression.

Non-viral plasmid delivery provides a more flexible approach to Klotho gene transfer. Kidney-targeted poly(dopamine)-polyethyleneimine-L-serine-Klotho plasmid nanoparticles (PPSK NPs) were designed to deliver Klotho plasmids to KIM-1-positive injured tubular epithelial cells, thereby linking Klotho expression to sites of renal injury [65]. This type of design is important because it addresses both Klotho restoration and disease-site selectivity. Rather than relying only on systemic exposure, injury-responsive or organ-targeted carriers may improve local therapeutic relevance and reduce unnecessary exposure in non-target tissues.

Physical-field-assisted gene delivery offers another strategy for spatial control. Ultrasound-targeted microbubble destruction has been used to deliver β-Klotho plasmid to the heart, enhancing FGF21 sensitivity and attenuating post-myocardial infarction cardiac remodeling [66,67]. This approach illustrates how external energy can be combined with carrier design to guide transgene delivery to a defined organ. For cardiovascular applications, such spatial control may be valuable when the therapeutic goal is local pathway modulation rather than systemic Klotho replacement.

mRNA/LNP-based Klotho delivery occupies an intermediate position between recombinant protein therapy and long-duration gene expression. Compared with DNA- or viral-vector-based approaches, mRNA delivery does not require genomic entry and generally supports transient, repeatable, and dose-adjustable expression. The key translational questions for mRNA/LNP systems are whether expression can be controlled within a safe pharmacodynamic window, whether repeated dosing remains tolerable, and which biomarkers best reflect target engagement.

### 5.2. Extracellular Vesicles and Cell-Derived Delivery Systems

Extracellular vesicles provide a biologically compatible strategy for transporting Klotho in a membrane-associated or cargo-protected form. Urine-derived extracellular vesicles carrying Klotho improved renal functional recovery in an acute tubular injury model, while Klotho-enriched mesenchymal stem cell-derived small extracellular vesicles promoted repair in acute kidney injury models [8,62]. These findings suggest that EV-based delivery may be particularly relevant for kidney disease, where Klotho deficiency, tubular injury, and renal repair are closely connected.

The potential value of EVs lies not only in cargo transport but also in biological context. Vesicle membranes may protect Klotho from rapid degradation, facilitate interaction with recipient cells, and support tissue-compatible delivery. In MSC-derived EV systems, Klotho delivery may also be combined with the anti-inflammatory, anti-apoptotic, and regenerative effects of the stem cell secretome. This makes EV-based strategies especially attractive for acute kidney injury and tissue repair, where therapeutic benefit may require coordinated biological effects rather than single-pathway intervention. For translation, however, EV-based Klotho products require clear product definition.

### 5.3. Recombinant Protein Delivery and Sustained-Release Formulations

Direct administration of recombinant soluble α-Klotho remains the most straightforward replacement strategy because the active molecule can, in principle, be manufactured, characterized, dosed, and monitored as a defined biologic product. Continuous intraperitoneal infusion using mini osmotic pumps maintained stable Klotho exposure in chronic kidney disease models and improved renal and cardiac outcomes [9]. This provides important pharmacological evidence that sustained soluble α-Klotho exposure can be beneficial. However, mini osmotic pumps are best viewed as experimental systems for exposure control rather than broadly applicable clinical delivery platforms.

Sustained-release biomaterials provide a more clinically adaptable route for local or regional Klotho delivery. Thermosensitive chitosan hydrogels have been used to achieve sustained local Klotho release in renal fibrosis models, while 3D-printed SilMA-based hydrogels enabled local delivery of soluble α-Klotho and mesenchymal stem cells for myotendinous junction regeneration [63,64]. These systems address the problem of local retention by maintaining Klotho near the disease site instead of relying on systemic distribution. They may be particularly useful for fibrosis, wound repair, musculoskeletal injury, and regenerative medicine applications.

From a formulation perspective, recombinant Klotho delivery also motivates the development of stabilizing excipients, lyophilized preparations, depot formulations, and long-acting protein formats. These approaches aim to preserve Klotho conformation, maintain biological potency, extend exposure, and improve administration convenience. Thus, sustained-release formulation should be viewed not only as a delivery strategy but also as part of the broader effort to convert soluble α-Klotho into a practical chronic biologic therapy.

### 5.4. Route-Oriented Strategies for Clinical Usability

Route convenience is especially important for Klotho-based therapeutics because many potential indications, including chronic kidney disease, neurodegeneration, cardiovascular aging, and health-span-associated applications, may require repeated or long-term administration. Subcutaneous long-acting formulations are therefore a particularly attractive direction. If combined with protein engineering or depot formulation, subcutaneous delivery could support outpatient administration while avoiding the complexity of continuous infusion.

For neurodegenerative and cognitive indications, central nervous system exposure remains a major challenge. Intranasal or nose-to-brain delivery is conceptually attractive because it may provide partial access to the CNS through anatomical pathways that reduce reliance on systemic circulation. This route may be useful when peripheral Klotho exposure is insufficient to achieve central target engagement. However, intranasal Klotho delivery still requires optimization of formulation stability, mucosal absorption, CNS biodistribution, and pharmacodynamic readouts.

Oral delivery represents a longer-term formulation frontier for Klotho and other protein therapeutics. Advances in oral peptide and protein delivery, including permeation enhancers, protective formulations, and lipid-based systems, provide useful conceptual guidance [68,69]. For Klotho, successful oral delivery would substantially improve patient acceptability and scalability for chronic use. Nevertheless, because Klotho is a large and structurally complex protein, oral delivery should currently be positioned as a future formulation direction rather than an established near-term route.

Overall, current Klotho delivery and formulation strategies have already addressed several key translational problems. Viral vectors provide sustained expression for long-term proof-of-concept studies. Non-viral nanoparticles and ultrasound-assisted systems improve tissue-selective or spatially guided delivery. mRNA/LNP platforms offer transient and tunable endogenous expression. EVs provide biologically compatible transport and renal repair potential. Recombinant protein infusion demonstrates the pharmacological value of sustained soluble Klotho exposure. Hydrogels enable local retention and programmable release. Route-oriented strategies, including subcutaneous, intranasal, and eventually oral delivery, aim to improve long-term usability. Future development should build on these strengths by matching each delivery strategy to the disease context, desired exposure duration, and pharmacodynamic readouts.

## 6. Molecular Engineering of Klotho: Improving the Drug Before Improving the Carrier

A key revision to the original framing is that delivery systems should not be expected to compensate for every weakness of the native molecule. For many biologics, molecular engineering precedes formulation optimization. Klotho should be approached similarly. Candidate engineering strategies include Fc fusion, albumin-binding domains, PEGylation, glycosylation optimization, protease-resistant variants, stabilized domains, and minimal functional peptides. Each strategy must be evaluated not only for half-life extension but also for preservation of receptor binding, pathway selectivity, and safety.

The Klotho-derived KP1 peptide illustrates the potential value of reductionist engineering. By retaining a defined anti-fibrotic mechanism through interference with TGF-β signaling, KP1 may offer advantages in manufacturing and formulation over full-length Klotho while potentially limiting broader systemic effects, including those related to mineral metabolism. As with other peptide therapeutics, however, KP1 may face inherent developability challenges, including limited oral bioavailability and rapid systemic clearance, which could constrain its clinical utility and will require careful evaluation during development. In vivo studies in mouse models of renal fibrosis have shown that intravenous KP1 preserved renal function, reduced fibrotic lesions, and increased endogenous Klotho expression, with preferential accumulation in injured kidneys [70]. However, KP1 has not yet entered human clinical trials, and its pharmacokinetic and safety profiles in humans remain undefined. Importantly, KP1 should not be viewed as a functional substitute for full-length Klotho. Rather, it represents a functionally selective peptide that retains anti-fibrotic and anti-senescence activities through TβR2/TGF-β blockade and posttranscriptional restoration of Klotho via the miR-223-3p/lncRNA-TUG1 axis, while its effects on FGF23-mediated mineral metabolism remain to be systematically characterized [70,71]. This example highlights the possibility of separating Klotho biology into therapeutically addressable functional domains. Accordingly, fibrosis, neuroprotection, mineral metabolism, and metabolic regulation may ultimately require distinct Klotho-derived or Klotho-mimetic products rather than a single replacement strategy intended to reproduce the full spectrum of Klotho functions.

Engineering also creates new assay requirements. A modified Klotho product should be characterized by identity, purity, glycosylation, higher-order structure, receptor or ligand binding, pathway-specific potency, stress stability, aggregation propensity, and immunogenicity risk. For fusion proteins or peptides, receptor bias and loss of physiological regulatory feedback must be assessed. The field would benefit from a consensus assay panel that allows direct comparison of Klotho constructs across laboratories.

## 7. Disease- and Route-Specific Translational Design

Klotho product design should begin with the disease context. In kidney disease, the target organ is both a source and a clearance site of α-Klotho, making renal impairment a determinant of exposure and response. Local kidney-targeted nanoparticles, EVs, or sustained-release systems may therefore offer advantages over untargeted systemic dosing. Biomarkers should include serum and urinary Klotho, phosphate, FGF23, vitamin D metabolites, renal function indices, inflammatory markers, and fibrosis-related readouts.

For neurodegenerative disease, the major challenge is delivery across or around the blood–brain barrier. Systemic recombinant protein may not provide sufficient central nervous system (CNS) exposure unless peripheral mechanisms are adequate. Intranasal delivery, CNS-tropic vectors, muscle-to-CNS endocrine expression, or engineered transport strategies may be more plausible than conventional intravenous protein injection. Cognitive endpoints should be paired with target engagement biomarkers, imaging or fluid biomarkers, and safety monitoring for mineral and metabolic effects.

For cardiovascular and metabolic disease, α-Klotho and β-Klotho programs should be clearly separated. α-Klotho replacement may be relevant to vascular calcification, endothelial dysfunction, cardiac fibrosis, and renal–cardiac cross-talk. β-Klotho-targeting therapies more closely resemble metabolic receptor agonists, especially those linked to FGF21/FGFR1c or FGF19/FGFR4 biology [5,6,7]. Their endpoints should include lipid parameters, insulin sensitivity, hepatic fat, bile acid biology, and cardiovascular safety.

For healthy aging and longevity-associated interventions, the evidentiary bar should be highest. Participants may not have life-threatening disease, so tolerability, reversibility, repeated-dose safety, biomarker validity, and conservative benefit claims are essential. In this setting, transient and controllable modalities may be preferable to irreversible or long-duration expression platforms until safety is better established.

## 8. Translational Roadmap and Future Perspectives

**Figure 3:**
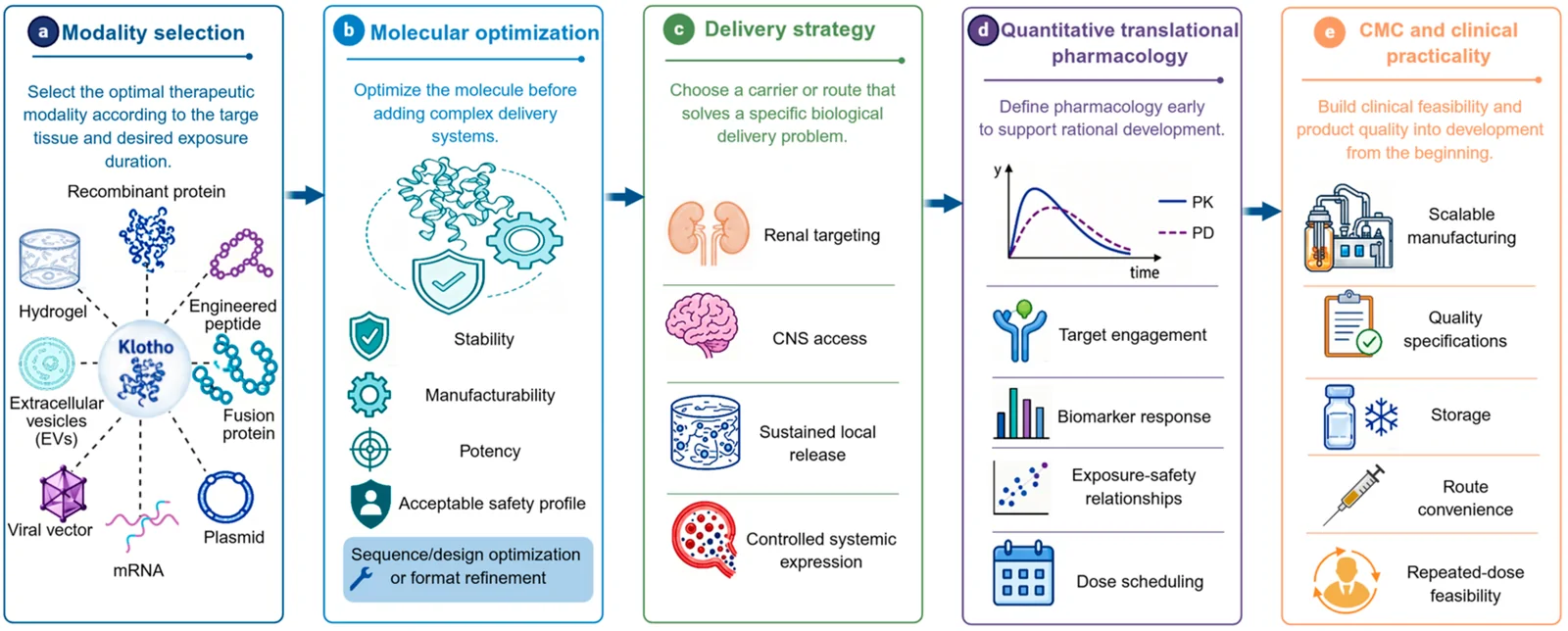
Translational roadmap for Klotho therapeutics. The roadmap integrates five key layers for Klotho therapeutic development: (**a**) modality selection, (**b**) molecular optimization, (**c**) delivery strategy, (**d**) quantitative translational pharmacology, and (**e**) CMC and clinical practicality. Created with BioRender.com.

A practical roadmap for Klotho therapeutics should integrate five layers (Figure 3). The first is modality selection, which should be guided by the target tissue, desired duration of exposure, and requirements for reversibility. These considerations necessitate balancing pharmacokinetic control, manufacturability, regulatory feasibility, and patient convenience. In this context, each delivery modality presents distinct advantages and limitations. Recombinant proteins offer precise dose titration but generally require frequent administration, whereas mRNA platforms enable transient and repeatable expression at the cost of demanding cold-chain logistics. Viral vectors can provide sustained expression, but their use is accompanied by concerns regarding immunogenicity and limited reversibility. EVs and hydrogels, in contrast, are attractive for localized delivery, yet their broader translation is currently constrained by limited regulatory precedents and challenges in scalable manufacturing.

The second layer focuses on molecular optimization. Before incorporating complex delivery systems, the therapeutic molecule itself should first be engineered to achieve an optimal balance among stability, manufacturability, potency, and safety. For protein-based therapeutics, this may involve Fc fusion or albumin-binding strategies to extend circulation half-life, glycoengineering to improve product homogeneity, and molecular minimization to identify functional peptide domains. For nucleic acid-based approaches, optimization strategies include codon adaptation, untranslated region engineering, and nucleoside modifications to enhance expression and reduce innate immune activation. Importantly, such modifications must be carefully evaluated in disease-relevant potency assays, as improvements in stability or pharmacokinetic properties may inadvertently compromise receptor interactions, alter pathway specificity, or introduce additional immunogenicity risks.

The third layer addresses delivery strategy. The delivery system or route should be selected to overcome a clearly defined biological barrier, such as achieving renal targeting, enabling CNS access, sustaining local exposure, or controlling systemic expression. For systemic protein delivery, formulation design must minimize rapid clearance and aggregation, whereas nucleic acid delivery requires protection from nuclease degradation, efficient cellular uptake and endosomal escape, and, where necessary, cell-type-specific targeting. For local applications, hydrogels and implantable systems can provide programmable release, but their development requires careful assessment of degradation products and sterilization processes. Importantly, the delivery strategy must also be compatible with the intended clinical setting, as outpatient injection, image-guided implantation, and continuous infusion differ substantially in terms of patient burden, compliance, and cost.

The fourth layer is quantitative translational pharmacology, which provides the basis for linking exposure to biological activity and clinical dosing. Pharmacokinetic and pharmacodynamic profiles, target engagement, biomarker responses, exposure-safety relationships, and dosing schedules should therefore be established early in development. Bioanalytical methods should distinguish exogenous from endogenous Klotho and quantify both total and functionally active concentrations. Because renal function may substantially influence α-Klotho clearance, pharmacokinetic studies in models of renal impairment are particularly important for informing dose selection in relevant patient populations. Pharmacodynamic biomarkers should capture both therapeutic activity, such as phosphate handling for α-Klotho and metabolic markers for β-Klotho, and on-target safety signals, including changes in phosphate, calcium, vitamin D, glucose, and lipid homeostasis, thereby enabling early detection of metabolic perturbations.

The fifth layer concerns CMC and clinical practicality, which should be incorporated from the outset rather than addressed only during late-stage development. Key considerations include scalable manufacturing, well-defined quality attributes, product stability and storage, administration convenience, and the feasibility of repeated dosing. For recombinant proteins, this requires robust cell line development, high-yield bioreactor processes, efficient downstream purification, and formulation optimization to minimize aggregation and chemical degradation. For mRNA/LNP systems, critical considerations include lipid quality, particle size, encapsulation efficiency, and mRNA integrity. Viral vectors require stringent control of empty-to-full capsid ratios and residual impurities, whereas EVs and hydrogels continue to face challenges in scalability and standardization. Establishing an early control strategy that links raw materials, in-process controls, release assays, and comparability protocols is therefore essential for managing process changes during scale-up and satisfying regulatory expectations. Looking ahead, AI-guided formulation optimization may further accelerate the development of robust delivery strategies for complex biologics such as Klotho [72].

Several near-term priorities emerge from this roadmap. First, recombinant and engineered Klotho proteins require systematic formulation stress testing together with stability-indicating potency assays to ensure that physicochemical stability is maintained without compromising biological activity. Second, gene- and mRNA-based strategies require quantitative control of transgene expression, along with predefined stopping criteria to limit excessive or prolonged exposure. Third, clinical trials should incorporate biomarkers that capture both the intended pharmacological effects and potential on-target safety signals, particularly those related to mineral and metabolic homeostasis. Finally, as company pipelines and clinical-trial activities continue to evolve rapidly, maintaining up-to-date information on ongoing development programs will be essential for accurately assessing the translational landscape of Klotho therapeutics.

## 9. Conclusions

Klotho occupies a distinct position among aging-related therapeutic targets because its clinical translation depends largely on the development challenges associated with biologic and nucleic acid modalities rather than on strategies established for small-molecule interventions such as senolytics or NAD^+^ boosters. Key barriers include molecular stability, manufacturability, delivery, tissue exposure, immunogenicity, dose control, and long-term safety, all of which must be addressed in an indication- and modality-specific manner. The future development of Klotho therapeutics is therefore unlikely to follow a single pathway. β-Klotho-directed biologics may offer opportunities to modulate metabolic signaling, whereas α-Klotho mRNA or gene-based approaches may enable sustained or tunable expression. Engineered soluble α-Klotho proteins and peptides may provide more conventional biologic development routes, while EVs, hydrogels, and related delivery systems could support localized or organ-targeted applications. Ultimately, the most promising strategies will be those that match the therapeutic modality to a well-defined clinical need and address a specific translational barrier rather than simply increasing Klotho exposure. By integrating molecular engineering, rational delivery design, quantitative pharmacology, biomarker-guided development, and robust CMC strategies, Klotho-based interventions may progress from compelling biological concepts toward more clinically tractable therapeutic approaches.

## Figures and Tables

**Table 4:** Representative Klotho delivery and formulation strategies in preclinical and translational studies.

Klotho Form	Delivery Strategy	Disease Model (Administration Route)	Release Feature	Key Advantages	Limitations
Membrane-bound α-Klotho	Recombinant adeno-associated virus carrying full-length membrane-bound α-Klotho cDNA [60]	Streptozotocin-induced diabetic nephropathy model (tail vein injection)	Sustained expression for at least 12 weeks	Long-term renal Klotho restoration, reduced hypertrophy and fibrosis via ROCK inhibition	Irreversible expression, immunogenicity risk, complex manufacturing and quality control
	Lentiviral vector-mediated α-Klotho upregulation [61]	Senescence-accelerated mouse prone-8 model (lateral ventricle injection)	Sustained brain expression	CNS-directed neuroprotection, improved memory and reduced oxidative stress	Invasive CNS administration, vector safety and off-target concerns
Soluble α-Klotho	Continuous recombinant soluble α-Klotho infusion using Alzet mini osmotic pump [9]	Chronic kidney disease model (continuous i.p. infusion)	Stable systemic exposure for approximately 12 weeks	Pharmacological proof-of-concept for sustained exposure, renoprotective and cardioprotective	Not patient-friendly, not feasible for chronic outpatient use
	Urine-derived extracellular vesicles naturally carrying Klotho [8]	Acute tubular injury model (i.v.)	Vesicle-associated Klotho cargo	Restored endogenous Klotho and renal function, biologically compatible	Variable cargo loading, scalable production and batch comparability not established
	Klotho-enriched mesenchymal stem cell-derived small extracellular vesicles [62]	Acute kidney injury model (i.v.)	EV-protected Klotho delivery	Combines Klotho with MSC regenerative and immunomodulatory effects	Product definition unclear, difficult to distinguish Klotho effects from carrier effects
	Thermosensitive chitosan hydrogel loaded with recombinant soluble α-Klotho [63]	Renal fibrosis model (in situ injection of renal capsule)	Local sustained release for approximately 14 days	Regional retention, attenuates renal fibrosis	Limited to local administration, not suitable for systemic indications
	3D-printed SilMA hydrogel with soluble α-Klotho + MSCs [64]	Myotendinous junction injury (local implantation)	Biphasic release	Anti-inflammatory and pro-regenerative in musculoskeletal repair	Requires surgical implantation, complex biomaterial characterization
	Kidney-targeted poly(dopamine)-polyethyleneimine-L-serine-Klotho plasmid nanoparticles [65]	AKI-to-CKD transition/tubular injury (i.v.)	Injury-site-selective renal gene delivery	Non-viral, disease-site-targeted, links expression to injury microenvironment	Preclinical only, targeting efficiency and long-term safety need further validation
	Lipid nanoparticle-formulated α-Klotho mRNA [21]	Clinical-stage platform/healthy volunteers (i.v.)	Transient and repeatable endogenous expression	Repeatable and reversible, dose-tunable, bridges protein and gene therapy	Requires cold-chain logistics, biomarker-guided dosing critical, clinical PK/PD data pending
β-Klotho plasmid	Ultrasound-targeted microbubble destruction with cationic microbubbles [66]	Post-myocardial infarction cardiac remodeling model (ultrasound-guided cardiac delivery)	Spatially guided plasmid delivery	Organ-targeted gene delivery, enhanced FGF21 sensitivity	Requires specialized ultrasound equipment, transient expression, limited to cardiovascular applications

## Data Availability

No new data were created or analyzed in this study. Data sharing is not applicable to this article.

## References

[1] Kuro-o M., Matsumura Y., Aizawa H., Kawaguchi H., Suga T., Utsugi T., Ohyama Y., Kurabayashi M., Kaname T., Kume E. (1997). Mutation of the mouse klotho gene leads to a syndrome resembling ageing. Nature.

[2] Buchanan S., Combet E., Stenvinkel P., Shiels P.G. (2020). Klotho, aging, and the failing kidney. Front. Endocrinol..

[3] Kuro-o M. (2019). The Klotho proteins in health and disease. Nat. Rev. Nephrol..

[4] Kuro-o M. (2010). Klotho. Pflügers Arch. Eur. J. Physiol..

[5] Kurosu H., Kuro-o M. (2008). The Klotho gene family and the endocrine fibroblast growth factors. Curr. Opin. Nephrol. Hypertens..

[6] Aaldijk A.S., Verzijl C.R.C., Jonker J.W., Struik D. (2023). Biological and pharmacological functions of the FGF19- and FGF21-coreceptor beta klotho. Front. Endocrinol..

[7] Lee S., Choi J., Mohanty J., Sousa L.P., Tome F., Pardon E., Steyaert J., Lemmon M.A., Lax I., Schlessinger J. (2018). Structures of β-klotho reveal a ‘zip code’-like mechanism for endocrine FGF signalling. Nature.

[8] Grange C., Papadimitriou E., Dimuccio V., Pastorino C., Molina J., O’Kelly R., Niedernhofer L.J., Robbins P.D., Camussi G., Bussolati B. (2020). Urinary extracellular vesicles carrying Klotho improve the recovery of renal function in an acute tubular injury model. Mol. Ther..

[9] Hu M.C., Shi M., Gillings N., Flores B., Takahashi M., Kuro-o M., Moe O.W. (2017). Recombinant alpha-Klotho may be prophylactic and therapeutic for acute to chronic kidney disease progression and uremic cardiomyopathy. Kidney Int..

[10] Hum J.M., O’Bryan L.M., Tatiparthi A.K., Cass T.A., Clinkenbeard E.L., Cramer M.S., Bhaskaran M., Johnson R.L., Wilson J.M., Smith R.C. (2017). Chronic hyperphosphatemia and vascular calcification are reduced by stable delivery of soluble Klotho. J. Am. Soc. Nephrol..

[11] Prud’homme G.J., Kurt M., Wang Q. (2022). Pathobiology of the Klotho antiaging protein and therapeutic considerations. Front. Aging.

[12] Zavvari Oskuye Z., Mehri K., Khalilpour J., Nemati S., Hosseini L., Bafadam S., Abdollahzade N., Badalzadeh R. (2025). Klotho in age-related cardiovascular diseases: Insights into mitochondrial dysfunction and cell death. Int. J. Cardiol. Heart Vasc..

[13] Hajare A.D., Dagar N., Gaikwad A.B. (2025). Klotho antiaging protein: Molecular mechanisms and therapeutic potential in diseases. Mol. Biomed..

[14] Yanucil C., Faul C. (2024). Klotho: A large protein with various beneficial functions, but also with therapeutic value?. J. Am. Soc. Nephrol..

[15] Minicircle (2026). Safety and Efficacy of Klotho and Follistatin Gene Therapy. https://ichgcp.net/clinical-trials-registry/NCT07285629.

[16] Minicircle (2026). Safety and Efficacy of Injectable Klotho Plasmid Gene Therapy in Humans. https://ichgcp.net/clinical-trials-registry/NCT07216781.

[17] CSPC Pharmaceutical Group (2024). CSPC’s Recombinant Fully Human Anti-βKlotho Monoclonal Antibody Approved for Clinical Trials. https://www.e-cspc.com/details/details_92_5394.html.

[18] eMedClub News (2025). Star Longevity Gene Therapy Company Klotho Expands its Footprint, Shifting the Anti-Aging Paradigm from Single-Disease Treatment to Systemic Intervention. https://www.sohu.com/a/917702764_120088173.

[19] Klotho Neurosciences (2025). US FDA Grants Orphan Drug Designation to Klotho Neurosciences’ KLTO-202 to Treat Amyotrophic Lateral Sclerosis. https://www.familydoctor.cn/news/meiguo-fda-shouyu-guer-zige-jiandongzheng-146770.html.

[20] Jocasta Neuroscience (2025). Jocasta Neuroscience Lands $35 Million Series a to Advance Longevity Protein Program for Managing Cognitive Impairment. https://www.familydoctor.cn/news/kasita-shenjingkexue-wanmeiyuan-a-tui-changshou-liaofa-177926.html.

[21] Klothea Bio Inc (2026). aKLmRNA-Mediated Protein Replacement Therapy. https://clinicaltrials.gov/study/NCT07544420.

[22] Saar-Kovrov V., Donners M.M.P.C., van der Vorst E.P.C. (2020). Shedding of Klotho: Functional implications in chronic kidney disease and associated vascular disease. Front. Cardiovasc. Med..

[23] Chen C.-D., Li Y., Chen A.K., Rudy M.A., Nasse J.S., Zeldich E., Polanco T.J., Abraham C.R. (2020). Identification of the cleavage sites leading to the shed forms of human and mouse anti-aging and cognition-enhancing protein Klotho. PLoS ONE.

[24] Chen L., Fu L., Sun J., Huang Z., Fang M., Zinkle A., Liu X., Lu J., Pan Z., Wang Y. (2023). Structural basis for FGF hormone signalling. Nature.

[25] Musgrove J., Wolf M. (2020). Regulation and effects of FGF23 in chronic kidney disease. Annu. Rev. Physiol..

[26] Prud’homme G.J., Wang Q. (2024). Anti-inflammatory role of the Klotho protein and relevance to aging. Cells.

[27] Donate-Correa J., Martín-Carro B., Cannata-Andía J.B., Mora-Fernández C., Navarro-González J.F. (2023). Klotho, oxidative stress, and mitochondrial damage in kidney disease. Antioxidants.

[28] Huang C.L. (2012). Regulation of ion channels by secreted Klotho. Adv. Exp. Med. Biol..

[29] Roig-Soriano J., Sánchez-de-Diego C., Esandi-Jauregui J., Verdés S., Abraham C.R., Bosch A., Ventura F., Chillón M. (2023). Differential toxicity profile of secreted and processed α-Klotho expression over mineral metabolism and bone microstructure. Sci. Rep..

[30] Vogt J., Föller M. (2025). Regulation of αKlotho. Cell. Physiol. Biochem..

[31] Al-Kadi A., Anter A., Rofaeil R.R., Sayed-Ahmed M.M., Ahmed A.-S.F. (2025). Klotho: A multifaceted protector in sepsis-induced organ damage and a potential therapeutic target. World J. Crit. Care Med..

[32] Kang J.S., Son S.S., Lee J.-H., Lee S.W., Jeong A.R., Lee E.S., Cha S.-K., Chung C.H., Lee E.Y. (2021). Protective effects of klotho on palmitate-induced podocyte injury in diabetic nephropathy. PLoS ONE.

[33] Lim S.W., Jin L., Luo K., Jin J., Shin Y.J., Hong S.Y., Yang C.W. (2017). Klotho enhances FoxO3-mediated manganese superoxide dismutase expression by negatively regulating PI3K/AKT pathway during tacrolimus-induced oxidative stress. Cell Death Dis..

[34] Mohammad G.H., Kieswich J., Kumar A., McCafferty K., Chakraborty R., Thiemermann C., Yaqoob M.M. (2025). Artesunate alleviates kidney fibrosis by restoring klotho protein and suppressing Wnt/β-catenin signalling pathway. Front. Cell Dev. Biol..

[35] Li S., Yu L., He A., Liu Q. (2019). Klotho inhibits unilateral ureteral obstruction-induced endothelial-to-mesenchymal transition via TGF-β1/Smad2/Snail1 signaling in mice. Front. Pharmacol..

[36] Mencke R., Olauson H., Hillebrands J.-L. (2017). Effects of Klotho on fibrosis and cancer: A renal focus on mechanisms and therapeutic strategies. Adv. Drug Deliv. Rev..

[37] Shaker M.R., Salloum-Asfar S., Taha R.Z., Javed I., Wolvetang E.J. (2025). Klotho overexpression protects human cortical neurons from β-amyloid induced neuronal toxicity. Mol. Brain.

[38] Zhao Y., Zeng C.-Y., Li X.-H., Yang T.-T., Kuang X., Du J.-R. (2020). Klotho overexpression improves amyloid-β clearance and cognition in the APP/PS1 mouse model of Alzheimer’s disease. Aging Cell.

[39] Dubal D.B., Yokoyama J.S., Zhu L., Broestl L., Worden K., Wang D., Sturm V.E., Kim D., Klein E., Yu G.-Q. (2014). Life extension factor klotho enhances cognition. Cell Rep..

[40] Daneshgar N., Dai D.-F., Grueter C.E. (2024). The aging heart: Exploring the role of Klotho in cardiac health/function. J. Cardiovasc. Aging.

[41] Palamar M., Grosu Radulescu I.D., Tanasescu M.D., Sircuta A., Bob F. (2025). Vascular calcification in chronic kidney disease and hemodialysis: Pathophysiological mechanisms and emerging biomarkers. Medicina.

[42] Edmonston D., Grabner A., Wolf M. (2024). FGF23 and klotho at the intersection of kidney and cardiovascular disease. Nat. Rev. Cardiol..

[43] Salah T.M., Rabie M.A., El Sayed N.S. (2025). Renoprotective effect of berberine in cisplatin-induced acute kidney injury: Role of Klotho and the AMPK/mtor/ULK1/Beclin-1 pathway. Food Chem. Toxicol..

[44] Wang S.-S., Zhang X., Ke Z.-Z., Zeng Y.-X., Wen X.-Y., Liu W.-B., Zhao J., Zhuang X.-D., Liao L.-Z. (2025). Klotho attenuates D-galactose-induced cardiac aging through the ROS/NLRP3/pyroptosis pathway. J. Mol. Cell. Cardiol..

[45] Li X.B., Liu J.L., Zhao S., Li J., Zhang G.-Y., Tang Q., Chen W.Y. (2025). Recombinant Klotho protein protects pulmonary alveolar epithelial cells against sepsis-induced apoptosis by inhibiting the Bcl-2/Bax/caspase-3 pathway. Adv. Clin. Exp. Med..

[46] Meroni M., Dongiovanni P., Tiano F., Piciotti R., Alisi A., Panera N. (2024). β-Klotho as novel therapeutic target in Metabolic Dysfunction-Associated Steatotic Liver Disease (MASLD): A narrative review. Biomed. Pharmacother..

[47] van Loon E.P.M., Pulskens W.P., van der Hagen E.A.E., Lavrijsen M., Vervloet M.G., van Goor H., Bindels R.J.M., Hoenderop J.G.J. (2015). Shedding of klotho by ADAMs in the kidney. Am. J. Physiol. Ren. Physiol..

[48] Zhao X., Han D., Zhao C., Yang F., Wang Z., Gao Y., Jin M., Tao R. (2024). New insights into the role of Klotho in inflammation and fibrosis: Molecular and cellular mechanisms. Front. Immunol..

[49] Ogawa Y., Kurosu H., Yamamoto M., Nandi A., Rosenblatt K.P., Goetz R., Eliseenkova A.V., Mohammadi M., Kuro-o M. (2007). BetaKlotho is required for metabolic activity of fibroblast growth factor 21. Proc. Natl. Acad. Sci. USA.

[50] Suzuki M., Uehara Y., Motomura-Matsuzaka K., Oki J., Koyama Y., Kimura M., Asada M., Komi-Kuramochi A., Oka S., Toru T. (2008). betaKlotho is required for fibroblast growth factor (FGF) 21 signaling through FGF receptor (FGFR) 1c and FGFR3c. Mol. Endocrinol..

[51] Hu M.C., Shi M., Zhang J., Addo T., Cho H.J., Barker S.L., Ravikumar P., Gillings N., Bian A., Sidhu S.S. (2016). Renal production, uptake, and handling of circulating alphaKlotho. J. Am. Soc. Nephrol..

[52] Zhong X., Jagarlapudi S., Weng Y., Ly M., Rouse J.C., McClure K., Ishino T., Zhang Y., Sousa E., Cohen J. (2020). Structure-function relationships of the soluble form of the antiaging protein Klotho have therapeutic implications for managing kidney disease. J. Biol. Chem..

[53] Cleland J.L., Lam X., Kendrick B., Yang J., Yang T.-H., Overcashier D., Brooks D., Hsu C., Carpenter J.F. (2001). A specific molar ratio of stabilizer to protein is required for storage stability of a lyophilized monoclonal antibody. J. Pharm. Sci..

[54] Sarsarshahi S., Bhattacharya S., Zacharias Z.R., Kamel E.S., Houtman J.C.D., Nejadnik R. (2025). Highly variable aggregation and glycosylation profiles and their roles in immunogenicity to protein-based therapeutics. J. Pharm. Sci..

[55] Eshraghi J., Galangashi R.B., Veilleux J.-C., Shi G., Collins D., Yang D., Vlachos P.P. (2025). Subvisible particle formation under mechanical and interfacial stress: A case study using bovine serum albumin as a model for biotherapeutic proteins. Int. J. Pharm..

[56] Kannan A., Shieh I.C., Negulescu P.G., Chandran Suja V., Fuller G.G. (2021). Adsorption and aggregation of monoclonal antibodies at silicone oil-water interfaces. Mol. Pharm..

[57] Manning M.C., Holcomb R.E., Payne R.W., Stillahn J.M., Connolly B.D., Katayama D.S., Liu H., Matsuura J.E., Murphy B.M., Henry C.S. (2024). Stability of protein pharmaceuticals: Recent advances. Pharm. Res..

[58] Moussa E.M., Panchal J.P., Moorthy B.S., Blum J.S., Joubert M.K., Narhi L.O., Topp E.M. (2016). Immunogenicity of therapeutic protein aggregates. J. Pharm. Sci..

[59] Matsuda T., Matsubara T., Hino S. (2006). Immunogenic and allergenic potentials of natural and recombinant innocuous proteins. J. Biosci. Bioeng..

[60] Deng M., Luo Y., Li Y., Yang Q., Deng X., Wu P., Ma H. (2015). Klotho gene delivery ameliorates renal hypertrophy and fibrosis in streptozotocin-induced diabetic rats by suppressing the Rho-associated coiled-coil kinase signaling pathway. Mol. Med. Rep..

[61] Zhou H.-J., Zeng C.-Y., Yang T.-T., Long F.-Y., Kuang X., Du J.-R. (2018). Lentivirus-mediated klotho up-regulation improves aging-related memory deficits and oxidative stress in senescence-accelerated mouse prone-8 mice. Life Sci..

[62] Deng X.-H., Wu Z.-C., Sun Q., Huang L.-X., Xie Y.-C., Lou D.-X., Li C.-G., Liu X.-Q., Zhou Z.-R., Tian T. (2025). The effects of Klotho delivering mesenchymal stem cell-derived small extracellular vesicles on acute kidney injury. J. Nanobiotechnol..

[63] Li C., Wang S., Liao C., Li Y., Zhou Y., Wu H., Xiong W. (2024). An in situ sustained-release chitosan hydrogel to attenuate renal fibrosis by retaining Klotho expression. Biomater. Res..

[64] Sun Y., Sheng R., Cao Z., Liu C., Li J., Zhang P., Du Y., Mo Q., Yao Q., Chen J. (2024). Bioactive fiber-reinforced hydrogel to tailor cell microenvironment for structural and functional regeneration of myotendinous junction. Sci. Adv..

[65] Li H., Ouyang Y., Lv H., Liang H., Luo S., Zhang Y., Mao H., Chen T., Chen W., Zhou Y. (2025). Nanoparticle-mediated Klotho gene therapy prevents acute kidney injury to chronic kidney disease transition through regulating PPARalpha signaling in renal tubular epithelial cells. Biomaterials.

[66] Yue C., Li R., Li C., Yang T., Huang X., Lei R., Yan Y., Liu Y., Li Q., Yan Q. (2024). Ultrasound-targeted microbubble destruction technology delivering beta-klotho to the heart enhances FGF21 sensitivity and attenuates heart remodeling post-myocardial infarction. Int. J. Mol. Med..

[67] Cao S., Zhou Q., Chen J.-L., Jiang N., Wang Y.-J., Deng Q., Hu B., Guo R.-Q. (2017). Enhanced effect of nuclear localization signal peptide during ultrasound-targeted microbubble destruction-mediated gene transfection. Mol. Med. Rep..

[68] Mrsny R.J., Mahmood T.A. (2021). Re-assessing PK/PD issues for oral protein and peptide delivery. Pharmaceutics.

[69] Griffin B.T., O’Driscoll C.M. (2011). Opportunities and challenges for oral delivery of hydrophobic versus hydrophilic peptide and protein-like drugs using lipid-based technologies. Ther. Deliv..

[70] Yuan Q., Ren Q., Li L., Tan H., Lu M., Tian Y., Huang L., Zhao B., Fu H., Hou F.F. (2022). A Klotho-derived peptide protects against kidney fibrosis by targeting TGF-β signaling. Nat. Commun..

[71] Zhang X., Li L., Tan H., Hong X., Yuan Q., Hou F.F., Zhou L., Liu Y. (2024). Klotho-derived peptide 1 inhibits cellular senescence in the fibrotic kidney by restoring Klotho expression via posttranscriptional regulation. Theranostics.

[72] Basak S. (2026). Artificial intelligence and machine learning-guided bio-orthogonal engineering of smart soft polymeric nanocarriers for precision drug delivery and translational nanomedicine. Front. Soft Matter.

